# Group psychotherapy for HIV patients. A different approach

**DOI:** 10.1186/1742-4690-9-S1-P92

**Published:** 2012-05-25

**Authors:** Michele Battuello, Paolo Roma, Giovanna Celia

**Affiliations:** 1 University of Rome Sapienza, Italy. Sant'Andrea Hospital, Rome, Italy

## Introduction

HIV is often related to psychological distress, after the diagnosis and the beginning of HAART. Brief Psychological approaches are important but they give only support, enhancing the relationships of the patients. In many cases these relationships are disfunctional too. Psychotherapies don´t focus on the indidvidual´s autonomy but on the support from the others.

## Objectives

A brief-grouppsychotherapy focused on major objectives of each person but with the main common objective to enhance the psychological individual indipendence and to promove a self- maturation; understanding the disfunctional dynamics realized in the past that are enhanced by the HIV-status, to promove individual indipendence with the objective of focusing the good and valid relationships and change the disfunctional ones and to allow the person to be able to open to the world again.

## Method

A brief group psychotherapy, supportive but mostly expressive. a small group 3 male, 1 female: were choosen for group psychotherapy after 2-3 individual meetings. 16 psychotherapy meetings, weekly, lenghth 1 hour 40-45 minutes.

## Results

First time patients worked on their disfunctional affective part, that was pre-hiv. In a second time they worked promoting their possibility of indipendence to find their lost self esteem. in the last time they focused to improve their relationships where possible but mostly to think that they can go out alone from the darkness after HIV diagnosis.

## Conclusions

Quality of life of HIV patients can be focused on changing their disfunctional parts, first enhancing the process of indipendence and individual esteem of the person.

